# Visualizing Plant Disease Distribution and Evaluating Model Performance for Deep Learning Classification with YOLOv8

**DOI:** 10.3390/pathogens13121032

**Published:** 2024-11-22

**Authors:** Abdul Ghafar, Caikou Chen, Syed Atif Ali Shah, Zia Ur Rehman, Gul Rahman

**Affiliations:** 1 College of Information Engineering, Yangzhou University, Yangzhou 225009, China; ayanghafar783@gmail.com (A.G.); ziaurrahman4h@gmail.com (Z.U.R.); gulrahman1234@gmail.com (G.R.); 2 School of Engineering and Applied Sciences, Bahria University Islamabad, Islamabad P.O. Box 44000, Pakistan; welcomeatif@yahoo.com; 3 Detechpro LLC, Integrating Solutions, Newark, DE 19906, USA

**Keywords:** plants diseases, convolutional neural networks, YOLO v8, DarkNet, ResNet

## Abstract

This paper presents a novel methodology for plant disease detection using YOLOv8 (You Only Look Once version 8), a state-of-the-art object detection model designed for real-time image classification and recognition tasks. The proposed approach involves training a custom YOLOv8 model to detect and classify various plant conditions accurately. The model was evaluated using a testing subset to measure its performance in detecting different plant diseases. To ensure the model’s robustness and generalizability beyond the training dataset, it was further tested on a set of unseen images sourced from Google Images. This additional testing aimed to assess the model’s effectiveness in real-world scenarios, where it might encounter new data. The evaluation results were auspicious, demonstrating the model’s capability to classify plant conditions, such as diseases, with high accuracy. Moreover, the use of YOLOv8 offers significant improvements in speed and precision, making it suitable for real-time plant disease monitoring applications. The findings highlight the potential of this methodology for broader agricultural applications, including early disease detection and prevention.

## 1. Introduction

Plant disease recognition is a critical field in agriculture with the potential to improve crop yield and reduce economic losses. If such issues not detected earlier, then it brings about the loss of thousands crop yield. The earlier detection of such diseases saves us from such losses. Deep learning techniques have proven successful in achieving accurate plant disease recognition. An essential step for developing and deploying a deep learning model for this task is data visualization. Visualization helps us to understand the data distribution, identify potential biases, and explore relationships between features.

This paper proposes a methodology to visualize plant disease recognition using YOLOv8 (You Only Look Once version 8). The dataset is sourced from Kaggle [1]. YOLOv8 is a state-of-the-art real-time object detection model well suited for tasks like image classification and recognition. The dataset utilized in this study consists of images depicting plants alongside their corresponding conditions, which include healthy, powdery, and rusty classifications. Prior to model training, the dataset was meticulously partitioned into distinct subsets for training and testing purposes, ensuring robust model evaluation. The custom-trained YOLOv8 model was rigorously assessed using the testing subset to gauge its performance across various conditions. Furthermore, to validate the model’s generalizability and effectiveness beyond the confines of the training dataset, it was subjected to testing on a separate set of images sourced from Google Images. The results of this evaluation were highly promising, demonstrating the model’s capability to accurately classify plant conditions even when presented with previously unseen data.

### Contribution

The paper presents innovative methodology and enhancements to the current state-of-the-art model to boost its efficiency and productivity.

Development of a Novel Methodology: The paper proposes a new methodology for plant disease detection using YOLOv8, a state-of-the-art real-time object detection model.

Custom Training of YOLOv8 Model: The model was custom-trained specifically for the task of detecting and classifying various plant conditions, such as diseases.

Comprehensive Evaluation: The paper rigorously evaluates the YOLOv8 model using a testing subset to assess its performance in detecting plant diseases under different conditions.

Generalization Testing: To validate the model’s robustness and generalizability, it was tested on previously unseen images from Google Images, simulating real-world scenarios.

Real-Time Application Potential: The use of YOLOv8 demonstrates significant improvements in both speed and accuracy, making it suitable for the real-time monitoring of plant diseases.

Broader Agricultural Impact: The findings suggest the potential for broader applications in agriculture, particularly for the early detection and prevention of plant diseases.

## 2. Literature Review

Plant diseases significantly impact crop yield and quality, and early detection is crucial for effective disease management. In recent years, deep learning techniques have shown promise in automating the detection and classification of plant diseases [2]. This literature review aims to provide a comprehensive evaluation of various deep learning models for plant disease detection and to identify potential knowledge gaps and future research directions.

In recent years, the field of plant disease detection has seen significant advancements, with deep learning techniques playing a crucial role [3]. Convolutional neural networks (CNNs) have emerged as the preferred method for this task, owing to their ability to effectively extract and classify visual features [4]. Among the various CNN architectures, the YOLO (You Only Look Once) family of models has gained considerable attention for the models’ real-time object detection capabilities [5]. YOLO8, the latest iteration of the YOLO (You Only Look Once) model, promises improved accuracy and efficiency in plant disease detection. To evaluate the performance of YOLO8 in this domain, a comparative analysis with other prominent CNN models, such as DarkNet, SqueezeNet, ResNet, and ResNet34, is essential. DarkNet [6], the underlying architecture of the YOLO models, has demonstrated strong performance in object detection tasks, making it a relevant benchmark. SqueezeNet, on the other hand, is known for its compact size and efficiency, making it a suitable candidate for deployment in resource-constrained environments. ResNet [7] and its variant, ResNet34, have gained widespread recognition for their ability to effectively capture complex visual features, which could be particularly relevant in the context of plant disease detection. By conducting a comprehensive comparison of YOLO8 [8] against these models, researchers can gain valuable insights into the strengths and limitations of each approach, ultimately informing the selection of the most suitable deep learning solution for plant disease identification [9].

Alam et al. [6] conducted a study on plant disease classification using the Xception CVPR 2017 architecture trained with the Adam optimizer. The results revealed that the Xception architecture achieved the highest validation accuracy and F1-score, indicating its superiority over previous approaches. The study suggested that this method could be extended to other agricultural applications, emphasizing its potential for transparent detection and classification purposes [10,11]. A study by Sun et al. [12] evaluated the performance of DenseNet-121 in leaf disease detection in crops. The findings demonstrated that DenseNet-121 achieved higher classification accuracy compared to state-of-the-art models, highlighting its effectiveness in disease detection for agricultural applications.

Falaschetti et al. [13] reported that InceptionResNetV2 achieved the best results followed by XceptionNet, while Peyal et al. [14] found that Inception-V3 was the best-suited model for detecting tomato leaf diseases with the best accuracy. These findings underscore the significance of exploring different deep learning architectures for specific plant disease detection tasks [15,16]. Joshi et al. [17] investigated the performance of ResNet34 and ResNet-101 in detecting plant leaf diseases. Furthermore, he reported 99.40% accuracy for the ResNet34 model and obtained the highest classification results with 97.74% accuracy. These studies highlight the viability of ResNet models in detecting plant diseases [18].

Dezaki et al. [15] presented a comparative analysis of different deep learning techniques, including convolutional neural networks (CNNs), Bayesian Optimized Support Vector Machine (SVM), and Random Forest Classifier [15]. While the study did not specify the exact performance metrics, it provided insights into the comparative evaluation of these techniques for plant disease detection [15,19]. The effectiveness of pretrained Densenet-120 in classifying crop diseases was shown [20,21], achieving high precision, F-1 score, and accuracy. The study emphasized the significance of using a combination of CNN models with fine-tuning adjustments for disease classification [17,22].

While the literature provides valuable insights into the performance of various deep learning models for plant disease detection, several knowledge gaps and potential future research directions can be identified: comparison with traditional machine learning techniques: Manzoor et al. provided an investigative comparison between support vector machines and deep learning for plant disease detection [23]. Future research could further explore the comparative performance of deep learning models with traditional machine learning techniques [6,8,12,24]. Exploration of specific crop diseases: Several studies focused on the detection of diseases in specific crops such as rice, tomato, and guava [25]. Future research could investigate the performance of deep learning models across a wider range of crops and diseases [26].

The literature primarily focuses on the performance metrics of deep learning models [8,27]. Future research could delve into the interpretability and explainability of these models to enhance their practical utility in agriculture. The integration of IoT and cloud computing involves the utilization of artificial intelligence and the Internet of Things for crop disease detection. Future research could explore the integration of IoT and cloud computing with deep learning models for real-time disease monitoring and management [21].

In conclusion, the literature review has shed light on the effectiveness of various deep learning models for plant disease detection. However, further research is warranted to address the identified knowledge gaps and leverage the potential of deep learning in agricultural applications. Overall, the literature reviewed indicates significant advancements in plant disease detection using deep learning models. The comprehensive evaluation of different deep learning architectures and the identification of potential knowledge gaps provide valuable insights for further research in this domain.

## 3. Dataset

The dataset used in this study is available from Kaggle [28]. It contains 1530 images of plant leaves categorized into three classes: healthy, powdery mildew, and rust. The data are split into training (80%), testing (10%), and validation sets (10%). The training set has the most images (1200), followed by the validation set (170) and the testing set (160). Within each set, the number of images for each class is displayed using a bar chart. For instance, the training set shows 400 healthy plant images, 300 powdery mildew images, and 200 rust disease images.

### 3.1. Healthy Leaf

**Figure 1:**
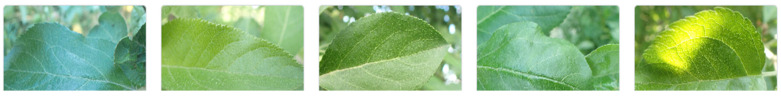
Healthy leaves; the figure shows a row of five images of healthy leaves. The leaves appear green and have various textures and lighting conditions.

An image of a healthy leaf would show a green leaf without any visible signs of disease. The leaf might have some veins or slight variations in color, but overall, it would appear healthy and undamaged. Figure 1 shows sample images for healthy leaves.

### 3.2. Powdery Mildew

**Figure 2:**
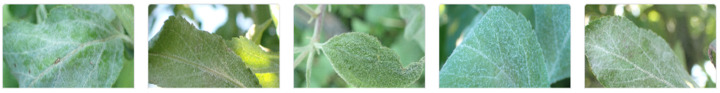
Powdery leaves; the figure shows a row of five images of powdery leaves. The leaves appear to have a white or gray powdery substance on their surfaces, indicating a potential disease or pest infestation.

An image of a powdery leaf would depict a leaf with white powdery spots on its surface. These spots are a characteristic symptom of powdery mildew, a fungal disease that affects plants, as shown in Figure 2. The severity of the disease might vary, with some leaves showing just a few spots and others being completely covered.

### 3.3. Rust Disease

**Figure 3:**
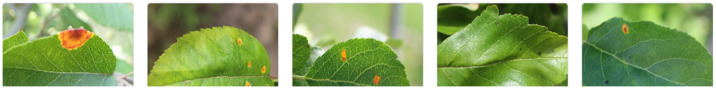
Rust; the figure shows a row of five images of leaves with rust. The leaves have orange or brown spots, characteristic of a plant disease caused by fungi.

An image of a rusty leaf would show a leaf with orange, brown, or yellow spots or pustules. These are signs of rust disease, caused by a different type of fungus. The spots might be distributed across the entire leaf or concentrated in specific areas; from Figure 3, we can see these spots.

## 4. Methodology

YOLOv8 is a single-stage object detection model. It predicts bounding boxes and class probabilities directly from input images in a single network. This makes it faster than traditional two-stage detection models that perform classification followed by bounding box regression. YOLOv8 offers several improvements over previous versions, including better accuracy and real-time inference capabilities.

For this study, we use YOLOv8 to visualize the distribution of images across the three disease classes within the training, testing, and validation sets. This visualization will help to identify any potential class imbalances in the data. The data are separated into three sections: a training set, testing set, and validation set. The training set has the most images (1200), followed by the validation set (300) and the testing set (130). Within each set, the number of images for each class is displayed using a bar chart. For instance, the training set shows 400 healthy plant images, 300 powdery mildew images, and 200 rust disease images.

**Figure 4:**
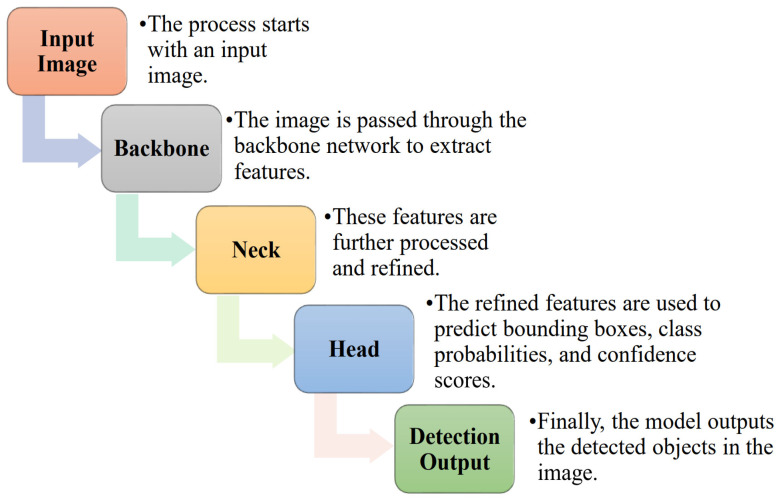
The figure is a diagram illustrating the architecture of the YOLO V8 object detection model. The model consists of three main components: the backbone, the neck, and the head. The input image is first passed through the backbone network to extract features. These features are then processed and refined in the neck. Finally, the head uses the refined features to predict bounding boxes, class probabilities, and confidence scores for the detected objects in the image.

The deep learning model employed in this study is based on the prebuilt YOLOv8 architecture, renowned for its state-of-the-art object detection capabilities characterized by high speed and accuracy. This model is fine-tuned using a proprietary dataset sourced from Kaggle, which consists of images depicting plants along with corresponding condition labels, encompassing healthy, powdery, and rusty conditions. YOLOv8 (You Only Look Once version 8) stands as a robust deep learning architecture dedicated to object detection tasks, renowned for its efficiency and speed. Rooted in the YOLO framework, this version introduces notable enhancements in architecture, training methodologies, and optimization techniques to achieve state-of-the-art performance in real-time object detection. Its backbone network, often based on DarkNet or CSP DarkNet, serves as a foundational feature extraction mechanism, efficiently capturing relevant features from input images. The detection head of YOLOv8 comprises convolutional layers meticulously designed to process feature maps and predict essential elements simultaneously. The basic process model of the desired framework is shown in Figure 4.

These predictions include bounding box coordinates relative to the feature map’s grid cells, confidence scores representing the probability of object presence, and class probabilities for each detected object. The integration of anchor boxes further refines the precision of bounding box predictions, with these predefined shapes adapting to the distribution of objects in the training dataset. Training YOLOv8 involves optimizing its parameters using variant loss functions that encompass localization loss, confidence loss, and classification loss. Through optimization algorithms such as stochastic gradient descent (SGD) or Adam, YOLOv8 fine-tunes its parameters to minimize the overall loss function, thereby enhancing its predictive capabilities. Throughout the training process, adjustments are made to the model’s parameters to enhance its efficacy in making precise predictions regarding the condition of plants. The model operates by receiving an image of a plant as input and subsequently providing a classification of the plant’s condition as output. Let us explore a simplified introduction to the DarkNet, SqueezeNet, ResNet, ResNet34, and YOLO V8 models to gain a better understanding of their key features and functionality.

Alex-Net: AlexNet is a deep convolutional neural network designed for image classification. It was a breakthrough in the field of deep learning, winning the ImageNet Large Scale Visual Recognition Challenge (ILSVRC) in 2012. AlexNet uses several convolutional layers to extract features from input images.

LeNet: LeNet is one of the earliest convolutional neural networks, designed for handwritten digit recognition (MNIST dataset).

DarkNet: DarkNet is the backbone network used in YOLO (You Only Look Once) for object detection. It mainly uses convolutional neural networks (CNNs).

SqueezeNet: SqueezeNet is a lightweight CNN designed to reduce the number of parameters while maintaining performance.

ResNet (Residual Network): ResNet introduces residual connections to help train very deep networks.

ResNet34: ResNet34 is a version of ResNet with 34 layers, using 3 × 3 convolutions and residual blocks.

These models all aim to make deep learning networks more efficient and effective by using specific structures like convolutions and residual connections to improve performance and training stability.

### YOLOv8 (You Only Look Once Version 8)

YOLOv8 is an advanced version of the YOLO family, designed for real-time object detection. It builds upon the concepts of earlier YOLO versions with enhancements in architecture and performance.

Backbone: The backbone network extract features from the input image. This is usually a convolutional neural network (CNN) like CSP DarkNet. The backbone processes the image through several layers to generate feature maps.

F = Backbone(I)

where I is the input image and F represents the feature maps.

Neck: The neck network further processes these feature maps to prepare them for the detection head. This might involve additional layers like the PANet (Path Aggregation Network) to enhance feature representation.

E = Neck(F)

where E are the enhanced feature maps.

Head: The detection head uses the processed feature maps to predict bounding boxes, object classes, and confidence scores. The output includes coordinates for bounding boxes, class probabilities, and confidence scores.

O = Head(E)

where O includes the bounding box coordinates, class probabilities, and confidence scores.

Loss Function: The training process involves a loss function that combines different types of losses, such as localization loss (for bounding box coordinates), confidence loss (for objectness score), and classification loss (for class prediction).

***Loss = λ_coord_L_coord_ + λ_conf_L_conf_ + λ_cls_L_cls_***

where *L_coord_* is the localization loss, *L_conf_* is the confidence loss, and *L_cls_* is the classification loss. *λ_coord_*, *λ_conf_*, and *λ_cls_* are the weights for each component.

Anchor Boxes: YOLOv8 uses anchor boxes to predict bounding boxes. These are predefined shapes that help the model detect objects of various sizes and aspect ratios. The model learns to adjust these anchors to fit the objects in the image.

Non-Maximum Suppression (NMS): After predicting multiple bounding boxes for each object, YOLOv8 applies NMS to eliminate redundant boxes. This ensures that each object is represented by only one bounding box with the highest confidence score.

B_final_ = NMS (B,threshold)

where B represents the predicted bounding boxes and threshold is the NMS threshold.

The above mathematical model provides a detailed explanation of the YOLOv8 object detection model. It describes the components of the model, including the backbone, neck, and head, as well as the techniques used for feature extraction, prediction, and loss calculation. Additionally, the images highlight the role of non-maximum suppression in eliminating redundant bounding boxes and the use of anchor boxes for object detection.

To adapt YOLOv8 specifically for plant disease detection, we implemented several customizations. First, we fine-tuned the model’s hyperparameters to optimize detection accuracy for the disease-specific features in our dataset. We also applied targeted data augmentation techniques, such as rotation, scaling, and color jittering, to enhance the model’s robustness to variations in leaf orientation and environmental lighting. Additionally, we customized anchor boxes within YOLOv8 to align with the typical sizes and shapes of disease patterns, such as spots and mildew patches. These adaptations help YOLOv8 effectively recognize and classify plant diseases, contributing a novel approach to real-time agricultural disease monitoring.

Hyperparameter Tuning: The hyperparameters of YOLOv8 were adjusted specifically for plant disease detection to optimize accuracy and reduce false positives. Key parameters, such as learning rate, batch size, and number of epochs, were fine-tuned based on validation performance to balance detection speed and accuracy. We found that a learning rate of 0.01, batch size of 16, and 50 epochs provided optimal performance for our dataset.

Data Augmentation: To increase model robustness, data augmentation techniques were applied to account for variations in lighting, orientation, and disease presentation. Augmentations included random rotations (up to 30 degrees), brightness and contrast adjustments, and color jittering to simulate different field conditions. This augmentation strategy allowed the model to generalize better to diverse real-world scenarios.

Anchor Box Customization: YOLOv8’s anchor boxes were customized to better capture the specific shapes and sizes of plant disease symptoms, such as irregular spots or mildew patches. By resizing anchor boxes to align with disease lesion characteristics, the model’s precision in detecting smaller and irregularly shaped areas of disease improved significantly. This customization enhances YOLOv8’s suitability for identifying plant diseases where symptoms are often non-uniform.

## 5. Results

**Figure 5:**
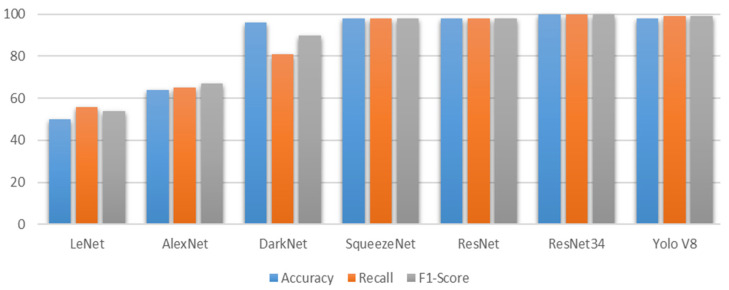
The figure is a bar graph comparing the performance of different neural network architectures (LeNet, AlexNet, DarkNet, SqueezeNet, ResNet, ResNet34, and YOLOv8) on average accuracy, recall, and F1-score. The results suggest that YOLOv8 generally outperforms the other models in terms of all three metrics, indicating its superior performance for the given task.

This section details the visualizations generated to understand the plant disease recognition dataset. These visualizations focus on depicting the distribution of images across the three disease classes (healthy, powdery mildew, and rust) within the training, testing, and validation sets. The detailed comparison of the implemented models is visually presented in Figure 5, allowing for a comprehensive analysis of their characteristics and performance.

### 5.1. Confusion Matrix

**Figure 6:**
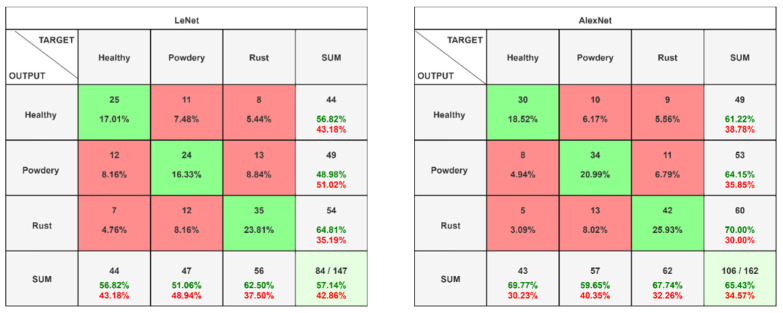

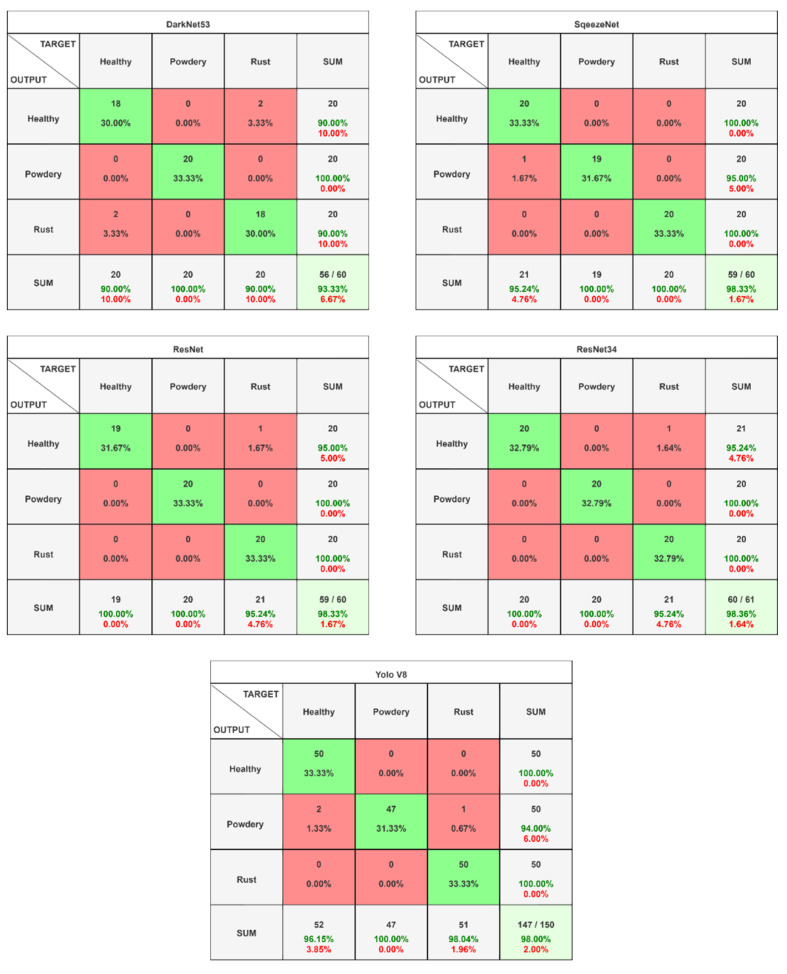
Confusion matrix of all models. The figure shows four confusion matrices, each representing the performance of a different model (LeNet, AlexNet, DarkNet, and SqueezeNet) on the task of leaf disease classification. The confusion matrices provide a detailed breakdown of the model’s predictions, including the number of correctly classified instances, false positives, and false negatives for each class (healthy, powdery, and rust). These matrices can be used to assess the model’s strengths and weaknesses and to identify areas for improvement.

The provided confusion matrix confirms a balanced distribution of images across the three classes within the training set. Each class (healthy, powdery mildew, rust) has roughly the same number of images (around 150). This is ideal for training a deep learning model, as the model will not be biased towards any particular class. The confusion matrix provides a summary of the predictions made by a model compared to the actual labels of the data. The F1–confidence curve allows one to visualize how the model’s performance changes as the confidence threshold is adjusted. The confusion matrix summary for all models is presented in Figure 6. The figure shows four confusion matrices, each representing the performance of a different model (LeNet, AlexNet, DarkNet, and SqueezeNet) on the task of leaf disease classification.

### 5.2. Model Performance

**Figure 7:**
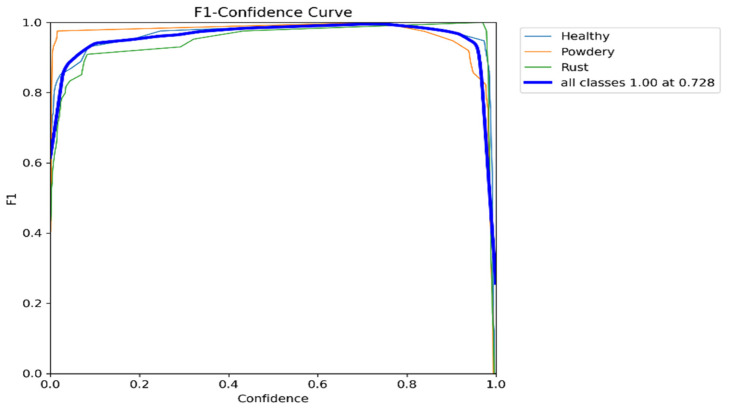
The figure is an F1–confidence curve, which illustrates the relationship between the F1-score and confidence for each class (healthy, powdery, and rust) in a multi-class classification problem. The curve shows how the F1-score varies as the confidence threshold is adjusted. The blue line represents the overall performance of the model across all classes, with an F1-score of 1.00 at a confidence threshold of 0.728.

The F1–confidence curve included in the image shows that the model achieves its maximum F1-score (1.0) at a confidence threshold of 0.728. This indicates that the model performs best when it predicts a disease class with at least 72.8% confidence. The curve also suggests that the model’s performance remains good even at lower confidence thresholds, as shown in Figure 7.

It is noteworthy that the confusion matrix and F1–confidence curve offer insights solely into the performance of the model on the training data. It is imperative to meticulously assess the model’s ability to generalize by scrutinizing its performance on unseen data from the testing and validation sets.

### 5.3. Sample Output

**Figure 8:**
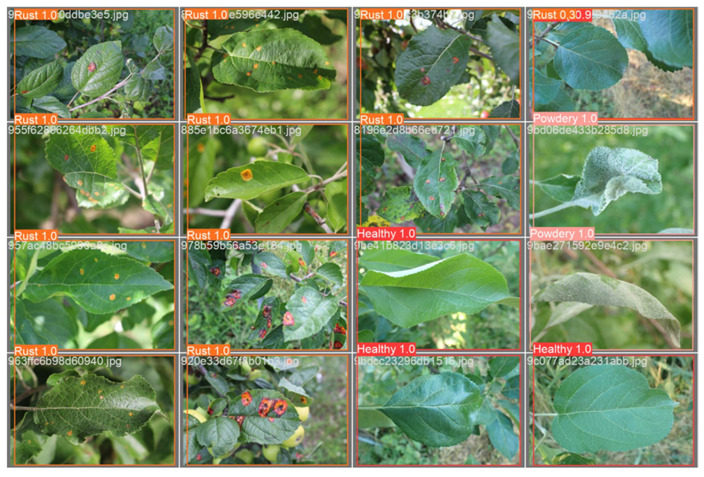
Sample output. The figure shows a grid of 16 images of leaves, each with a bounding box and a predicted class label (rust, healthy, or powdery). The bounding boxes indicate the location of the detected disease or healthy regions within the leaves. The predicted class labels suggest the model’s classification results for each image.

The illustration of Figure 8 depicts a grid of 16 leaf images, each featuring a bounding box and a predicted class label (rust, healthy, or powdery). The bounding boxes delineate the detected disease or healthy regions within the leaves, while the predicted class labels represent the model’s classification results for each image.

### 5.4. Results from Google Images

**Figure 9:**
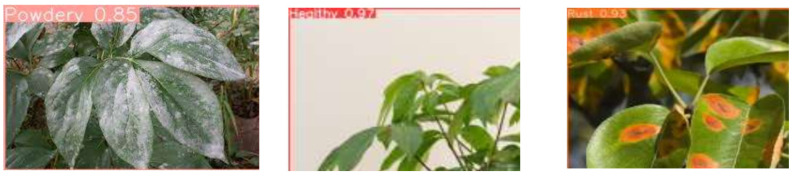
Images taken from Google. The figure shows a row of three images of leaves with different diseases. The first image shows a leaf with powdery mildew, the second shows a healthy leaf, and the third shows a leaf with rust. The predicted class labels and confidence scores for each image are also provided.

In Figure 9, there is a series of images sourced from Google displaying leaves affected by various diseases. The first image showcases a leaf with powdery mildew, the second reveals a healthy leaf, and the third presents a leaf with rust. Additionally, the figures include the predicted class labels and confidence scores for each image.

## 6. Discussion

This study presents a methodology using YOLOv8 for detecting plant diseases, advancing current capabilities in real-time image-based plant health monitoring. YOLOv8, a state-of-the-art model, showcases improved performance over previous YOLO versions and comparable object detection frameworks. Here, we discuss the implications of our findings in relation to the existing literature, the practical benefits for agricultural monitoring, limitations, and potential directions for future research.

### 6.1. Comparison with Prior Studies

Previous research has demonstrated the effectiveness of various convolutional neural networks (CNNs) like ResNet, DenseNet, and older YOLO versions (YOLOv3, YOLOv5) for plant disease detection. Previous studies demonstrated high accuracy using models like ResNet34 and DenseNet-121 for specific crop diseases, with each model showing strengths in certain areas of feature extraction and classification accuracy [17,29]. However, these models are not as optimized for real-time processing as YOLO-based architectures. By leveraging YOLOv8, which combines high detection speed with improved accuracy, our approach enables efficient, real-time plant disease monitoring. The model’s ability to handle large volumes of data swiftly without compromising accuracy places it ahead of many previously used CNN architectures in practical, on-field applications.

### 6.2. Practical Implications

The application of YOLOv8 in plant disease detection offers significant advantages in precision agriculture. The model’s real-time detection capabilities allow for rapid disease identification, which can facilitate timely intervention and minimize crop losses. This is particularly valuable in large-scale farming where manual inspection is infeasible. YOLOv8’s adaptability to various plant diseases, as demonstrated by our testing on both Kaggle-sourced and external datasets, suggests that this approach can be expanded to monitor other types of crops and diseases. By integrating such a model into existing agricultural monitoring systems, farmers and agronomists can make proactive, data-driven decisions to protect crops, optimize yield, and reduce economic losses associated with delayed disease detection.

### 6.3. Limitations

While YOLOv8 demonstrates considerable strengths, certain limitations should be noted. Our model was trained on a dataset with a limited range of plant diseases (healthy, powdery mildew, rust), which may restrict its generalizability to other disease types or crop varieties without further training. Additionally, though the model performs well in controlled testing scenarios, environmental factors such as lighting, image resolution, and occlusion in real-world settings could affect its accuracy. Future studies could address these limitations by expanding the dataset to include diverse crop types and disease conditions, as well as by testing in varied environmental settings.

## 7. Conclusions

This study demonstrates the viability of YOLOv8 for real-time plant disease detection, highlighting its potential to advance agricultural disease monitoring systems. By building on this foundation, future research can broaden the scope of plant disease detection, contributing to more resilient and sustainable agricultural practices. The dataset utilized in this study consists of images depicting plants alongside their corresponding conditions, which include healthy, powdery, and rusty classifications. Before model training, the dataset was meticulously partitioned into distinct subsets for training and testing purposes, ensuring robust model evaluation. The custom-trained YOLOv8 model was rigorously assessed using the testing subset to gauge its performance across various conditions. Furthermore, to validate the model’s generalizability and effectiveness beyond the confines of the training dataset, it was subjected to testing on a separate set of images sourced from Google Images. The results of this evaluation were highly promising, demonstrating the model’s capability to accurately classify plant conditions even when presented with previously unseen data.

Previous studies have shown the effectiveness of convolutional neural networks (CNNs) like ResNet34 and DenseNet-121 for plant disease detection. For instance, Joshi et al. achieved high accuracy using ResNet34 on specific crop diseases, demonstrating its strength in feature extraction and classification accuracy [17]. Similarly, Sun et al. found DenseNet-121 to perform exceptionally well for leaf disease detection due to its robust architecture and feature reuse capabilities [30]. However, these CNN architectures, while highly accurate, are typically more suited to static, batch-processing environments rather than real-time applications. In contrast, YOLO-based models, particularly YOLOv8, combine real-time detection speed with accuracy, enabling efficient plant disease monitoring directly in the field. This capability makes YOLOv8 advantageous for large-scale, real-world applications where immediate intervention is essential.

Future research can build upon this study by exploring several avenues. Expanding the dataset to include more disease classes and different crop types would enable the model to generalize better across agricultural domains. Additionally, integrating IoT and cloud computing with YOLOv8 could enhance real-time monitoring in resource-constrained environments. Advances in transfer learning and model compression techniques could further optimize YOLOv8 for use in mobile or remote sensing devices, increasing its accessibility for small-scale farmers and regions with limited computational resources. Exploring ensemble methods that combine YOLOv8 with other CNN architectures could also enhance model robustness and accuracy, particularly for more complex disease classifications.

## Data Availability

Data are contained within the article.
